# The Effect of Coronal Implant Design and Drilling Protocol on Bone-to-Implant Contact: A 3-Month Study in the Minipig Calvarium

**DOI:** 10.3390/ma14102645

**Published:** 2021-05-18

**Authors:** Omer Cohen, Dieter D. Bosshardt, Evegeny Weinberg, Gil Slutzkey, Ofer Moses

**Affiliations:** 1 Department of Periodontology and Dental Implantology, School of Dental Medicine, Tel-Aviv University, Tel-Aviv 6997801, Israel; evgenywein@gmail.com (E.W.); slutzkey@gmail.com (G.S.); mosesofer@gmail.com (O.M.); 2 Laboratory of Oral Histology, School of Dental Medicine, University of Bern, 3012 Bern, Switzerland; dieter.bosshardt@zmk.unibe.ch

**Keywords:** flutes, microthreads, drilling, bone-to-implant contact

## Abstract

**Background:** Stress concentrated at an implant’s neck may affect bone-to-implant contact (BIC). The objective of this study was to evaluate four different implant neck designs using two different drilling protocols on the BIC. **Methods:** Ninety-six implants were inserted in 12 minipigs calvarium. Implants neck designs evaluated were: type 1–6 coronal flutes (CFs), 8 shallow microthreads (SMs); type 2–6 CFs,4 deep microthreads (DMs); type 3–4 DMs; type 4–2 CFs, 8 SMs. Two groups of forty-eight implants were inserted with a final drill diameter of 2.8 mm (DP1) or 3.2 mm (DP2). Animals were sacrificed after 1 and 3 months, total-BIC (t-BIC) and coronal-BIC (c-BIC) were evaluated by nondecalcified histomorphometry analysis. **Results:** At 1 month, t-BIC ranged from 85–91% without significant differences between implant types or drilling protocol. Flutes on the coronal aspect impaired the BIC at 3 m. c-BIC of implant types with 6 CFs was similar and significantly lower than that of implant types 3 and 4. c-BIC of implant type 4 with SMs was highest of all implant types after both healing periods. **Conclusions:** BIC was not affected by the drilling protocol. CFs significantly impaired the -BIC. Multiple SMs were associated with greater c-BIC.

## 1. Introduction

Osseointegration has been defined as a direct and functional connection between a bone and an artificial implant [1]. Implant stability is a prerequisite for achieving osseointegration. Micromovements exceeding 50–100 µm may result in fibrous tissue formation instead of osseous integration [2]. Implant stability during the healing process is a result of primary and secondary stability. Primary stability depends on several factors, including bone density site dimensions, drill speed, and drill feed-rate during osteotomy preparation, surgical technique, and macro-/microscopic implant morphology [3,4,5]. Secondary stability is sequential to new bone formation and remodeling at the bone-implant interface. It is also dependent on site-specific bone quality, surgical osteotomy preparation, and implant design [6,7,8].

Initial primary stability stems from implant surface zones engaging in direct contact with the surrounding bone. This mechanical compression provides primary stability up to a certain point in time. Later, bone in direct contact with the implant will undergo remodeling, a process leading to increasing secondary stability. Void spaces between the implant and bone, which do not contribute to primary stability, are filled with a blood clot following implant placement; later, these clots are replaced gradually by mature bone [9].

Several studies have suggested that implants should be inserted using an increased torque to increase their primary stability, which may allow immediate loading [4,5]. To increase primary stability, it has been suggested that tapered implants should be inserted into osteotomy sites prepared with a final drill that is slightly narrower than the implant diameter [10]. However, high localized compression may lead to ischemia and eventually bone necrosis in close vicinity to the implant [11]. This phenomenon is generally considered to be limited to cortical bone prevailing in the crestal compartment of the alveolar process. High compression caused by an insertion torque higher than 40–45 Ncm has been claimed to compromise the local microcirculation, leading to bone necrosis and osteocyte death, followed by bone resorption [12].

While some studies have reported increased bone-to-implant contact (BIC) with increased insertion torque, others have reported a reduced BIC in implants installed with a high insertion torque. Duyck et al. [13] compared the BIC of implants inserted with a high torque (above 50 Ncm due to underdrilling) to that of implants inserted with a low torque (up to 10 Ncm due to overdrilling) in the rabbit tibia. After two weeks, implants inserted with a high torque demonstrated a significantly higher BIC than implants inserted with a low torque. At four weeks, no difference in the BIC was found between the groups [13]. In a canine model, 4.0-mm-diameter implants inserted into undersized 3.2-mm or 3.5-mm osteotomy sites presented extensive necrotic bone areas at the sites of the three coronal implant threads after one week. After three weeks, these regions showed bone remodeling with a restricted amount of new bone [14]. Implants inserted with a high torque (mean, 110 Ncm) in the sheep mandible, composed of thick (3–4 mm) cortical bone, demonstrated intrabony pockets around the implant neck as early as six weeks after placement [15]. Recently, it was reported that the insertion of implants with an overdrilling protocol for the crestal aspect of the osteotomy in a rabbit tibia model resulted in increased short-term crestal bone-to-implant contact [16]. Crestal bone comprises of cortical bone; hence, the coronal design of an implant can affect primary stability and crestal bone resorption [17].

Modifications to the implant macroscopic design and surface roughness are intended to allow the bone anchorage of dental implants and shorten the time required for osseointegration, if possible.

Flutes, which are located at the apex of implants, are intended to increase the self-tapping ability of the implant tip and facilitate implant insertion due to the cutting feature. The reported advantages of fluted implants include a decrease in the heat generated during insertion, insertion torque, operative duration, and the number of surgical instruments [18,19]. Historically, several types of flute designs have been used at the implant tip, such as edge, bowl, and spiral designs. In tapered implants, bowl flutes were reported as the optimal design, with a lower resistance to initial insertion and higher stability, for final instrumentation [20]. As implants are inserted, stress is concentrated on the coronal aspect. It has been previously reported that excessive stress can lead to bone resorption [21,22]. To distribute stress at the coronal aspect, microthreads were introduced. It is advocated that microthreads promote bone formation, distribute stress in the cancellous bone [23], and increase the maximal stress the cortical bone can sustain [24]. To date, no general agreement has been reached regarding the effectiveness and influence of the implant macro- or microscopic design on the bone-to-implant contact. The aim of this study was to evaluate the influence of four different coronal implant neck designs using two different drilling protocols on the BIC in the minipig calvarium.

## 2. Materials and Methods

Twelve female Sinclair minipigs, 7.5 months of age and 48–53 kg in weight, were used in the study. The number of animals was determined by considering previous studies, and performing a sample size calculation for a statistical power of 0.8 [25,26,27].

**Figure 1:**
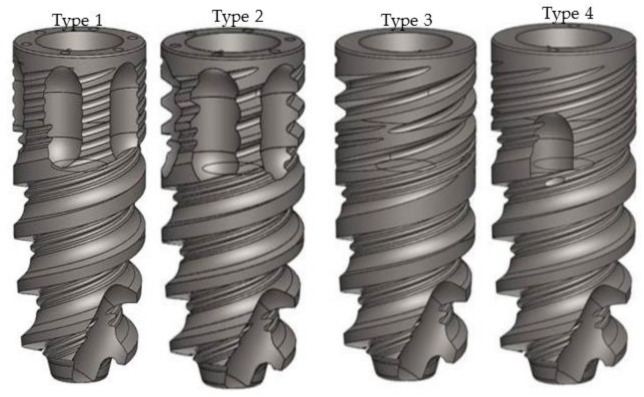
The four types of NeO ^®^ implants inserted: Type 1—implant with 6 coronal flutes and 8 shallow microthreads. Type 2—implant with 6 coronal flutes and 4 deep microthreads. Type 3—implant with 0 coronal flutes and 4 deep microthreads. Type 4—implant with 2 shorter coronal flutes and 8 shallow microthreads.

Ninety-six NeO ^®^ (Alpha Biotech, Petah-Tikva, Israel) implants (titanium grade, 23; Ra, 1.6 µm; Ø, 3.75/7.5 mm) with 4 different coronal designs, 24 implants of each type, were inserted in the parietal bone of the calvarium (Figure 1).

Type 1—NeO ^®^ implant with 6 coronal flutes (2.26 mm in length, 1.15 mm in width and 0.35 mm in depth) located 0.25 mm apically from the implant shoulder and 8 shallow microthreads (0.05 mm deep; pitch, 2.4 mm).

Type 2—NeO ^®^ implant with 6 coronal flutes (2.62 mm in length, 1.35 mm in width and 0.55 mm in depth) located 0.25 mm apically from the implant shoulder and 4 deepmicrothreads (0.3 mm deep; pitch, 2.4 mm).

Type 3—NeO ^®^ implant with 0 coronal flutes and 4 deep microthreads (0.3 mm deep).

Type 4—NeO ^®^ implant with 2 coronal flutes (1.4 mm in length, 1.15 mm in width and 0.35 mm in depth) located 1.32 mm apically from the implant shoulder and 8 shallow microthreads (0.05 mm deep; pitch, 2.4 mm).

Implants were inserted following preparation of the osteotomy by a drilling unit with 3–5 NCm steps (W&H, Elcomed, Burmous, Austria) with two different drilling protocols:

Drilling protocol 1: Underdrilling protocol (DP1). The osteotomy was prepared starting with a marking drill (Ø, 1.2 mm) followed by a second drill (Ø, 2.0 mm) and a final drill (Ø, 2.8 mm).

Drilling protocol 2: Standard drilling protocol (DP2) as suggested by the manufacturer. The osteotomy was prepared starting with a marking drill (Ø, 1.2 mm) followed by a second drill (Ø, 2.0 mm), a third drill (Ø, 2.8 mm) and a final drill (Ø, 3.2 mm).

Animals were sacrificed after 1 and 3 months of healing. The study protocol was approved by the ethics committee of Assaf-Harofeh Medical Center, Israel (45/2015). The study design followed the Animal Research Reporting In Vivo experiments (ARRIVE) guidelines [28]

### 2.1. Surgical Procedure

**Figure 2:**
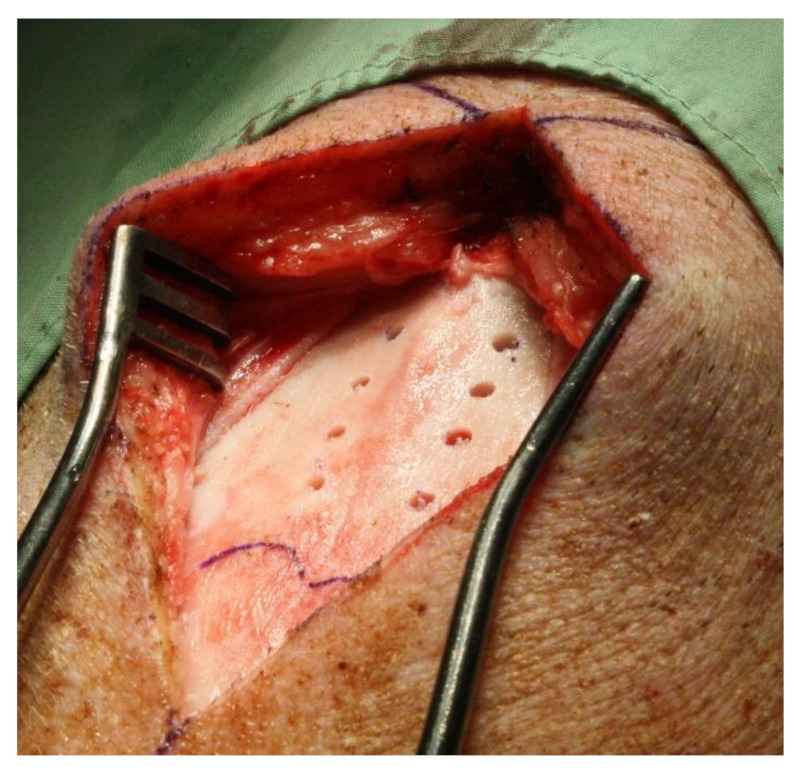
Calvarium after preparation of osteotomies with the standard underdrilling protocol as suggested by the manufacturer (DP2) and the extra underdrilling protocol (DP1).

All animals were premedicated with an intramuscular injection of 20 mg/kg ketamine (Ketalar, Pfizer, Herzliya, Israel) and 2 mg/kg xylazine (Sedaxylan, Eurovet Animal Health, Bladel, The Netherlands). Isoflurane 2% and oxygen were then used to maintain anesthesia. After animals were placed in a prone position, the surgical site was meticulously shaved, followed by scrubbing using povidone-iodine 10% (Teva Medical, Tel-Aviv, Israel) + chlorhexidine 4% (Vitamed, Binyamina, Israel) and draping to ensure sterility. Antibiotics (cefazolin, 1 g) and analgesics (7.5 mg of midazolam and 1000 mg of dipyrone) were administered before surgery. Analgesics were administered for an additional 3 days after surgery. A linear midline skin incision was made to expose the bone surface. Osteotomies were prepared lateral to the midline suture in the parietal bone apical to the supraoccipital protuberance and coronal to the frontoparietal suture. Four osteotomies were prepared according to DP1 (one side of the midline cranial suture, randomly assigned), whereas another 4 osteotomies were prepared according to DP2 (contralateral side of the midline suture) (Figure 2). Osteotomies were prepared with sharp drills and profuse irrigation with cold, sterile saline. The location of each implant was chosen randomly.

The submerged implants were sealed with a cover screw. Flaps were closed in layers with resorbable sutures (Vicryl, Ethicon, Cornelia, GA, USA) followed by suturing and wound closure.

### 2.2. Histological Processing and Analysis

Following euthanasia, samples were chemically fixed and prepared for the preparation of nondecalcified ground sections, as previously described [29,30]. Briefly, the samples were fixed by continuous submersion in 4% neutral buffered formaldehyde for 1 week at +4°C, dehydrated by continuous submersion in ascending concentrations of alcohol and finally cleared by xylol I and xylol II. Thereafter, the samples were embedded in methyl methacrylate (MMA). Polymerization was carried out for up to four days in sealed glass vials submerged in a water bath to assure constant room temperature without movement. The embedded tissue blocks were aligned according to µ-CT imaging [30] and depressions made on the implant platform shoulder above the flutes of implant types 1, 2, and 4 to cut through the center of two opposing flutes. Three sections were cut at a thickness of 500 µm using a low-speed diamond saw with coolant (Varicut^®^ VC-50, Leco, Munich, Germany). After mounting the sections onto acrylic glass slabs, they were ground and polished to a final thickness of approximately 100 µm (Knuth-Rotor-3, Struers, Rodovre/Copenhagen, Denmark) and stained with toluidine blue/McNeil combined with basic fuchsin [27]. Digital photography was performed using a digital camera (AxioCam MRc; Carl Zeiss, Gottingen, Germany) connected to a microscope (Axio Imager M2; Carl Zeiss, Gottingen, Germany).

The total bone-to-implant contact (t-BIC) and coronal bone-to-implant contact (c-BIC) were histomorphometrically evaluated in the central-most ground section of each implant.

**Figure 3:**
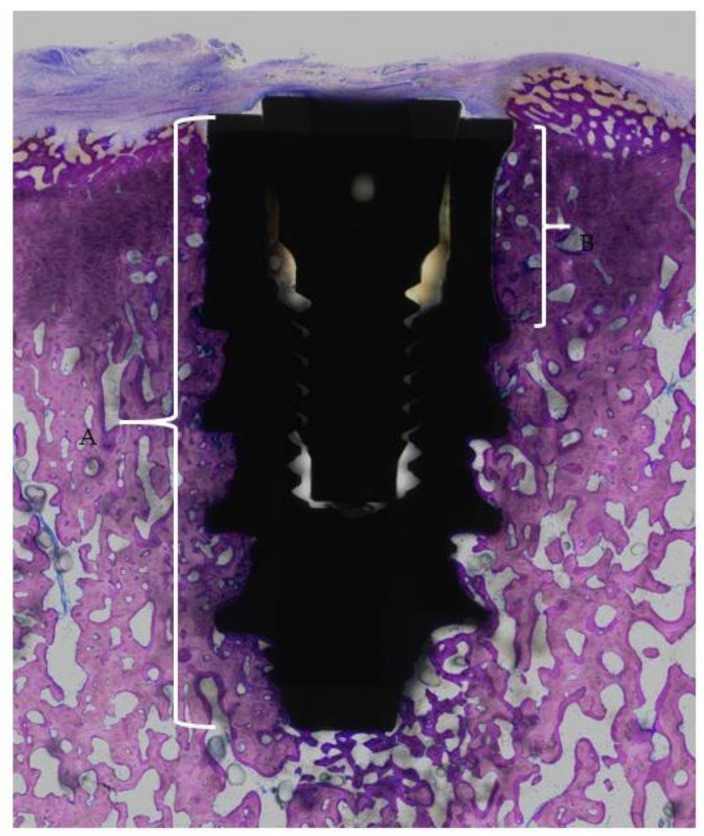
Micrograph of a whole implant. A represents the region of total bone-to-implant contact (t-BIC). B represents the region of the coronal third of cortical bone-to-implant contact (flute-BIC). Magnification, 10×.

For determination of the bone-to-implant contact (BIC), the following landmarks were identified on each ground section (Figure 3):

- The implant shoulder
- The apical end of the implant
- The coronal end of the flute
- The apical end of the flute

The histomorphometric analysis was performed as described by Janner et al. [31] R software v 3.3.3 (R Core Team 2013. Vienna, Austria) was used for statistical analysis of the data. A nonparametric test (Kruskal-Wallis) was used to evaluate differences among the implant types and between the drilling protocols. All *p* values were corrected for multiple testing by applying Bonferroni-Holm’s method. *p* < 0.05 was set as the significance level.

## 3. Results

**Figure 4:**
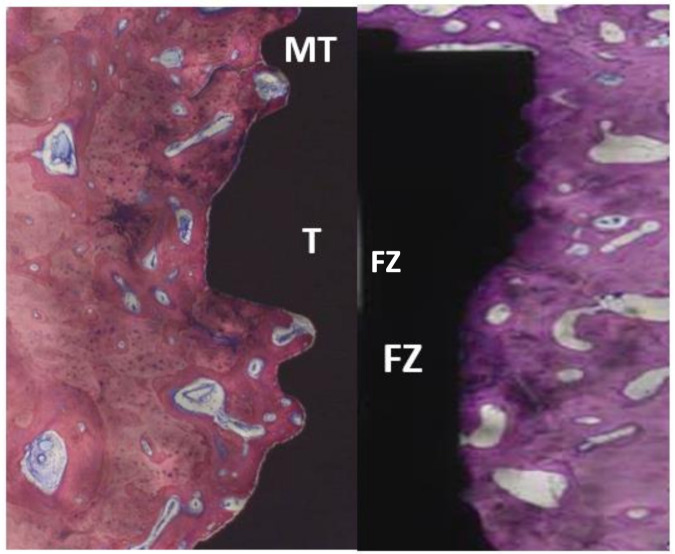
Typical micrographs of the coronal aspect of implants after 1 m and 3 m, revealing new bone close to the implant profile along with blood vessels with no signs of inflammation. MT, microthread; T, thread; FZ, flute zone.

**Figure 5:**
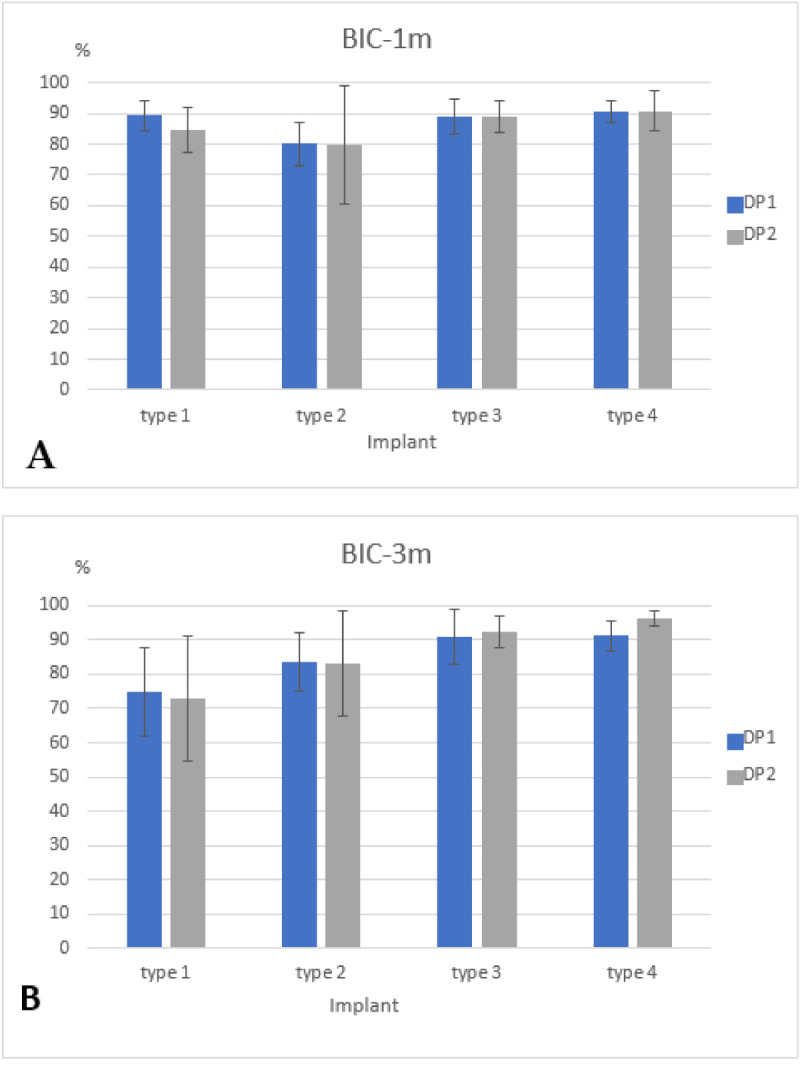
(**A**)—Total bone-to-implant contact one month after (t-BIC-1 m) implants were inserted with the standard underdrilling protocol (DP1) and the extra underdrilling protocol (DP2). (**B**)—Total bone-to-implant contact three months after (t-BIC-3 m) implants were inserted with the standard underdrilling protocol (DP2) and the extra underdrilling protocol (DP1). Data are presented as the average ± sd.

**Figure 6:**
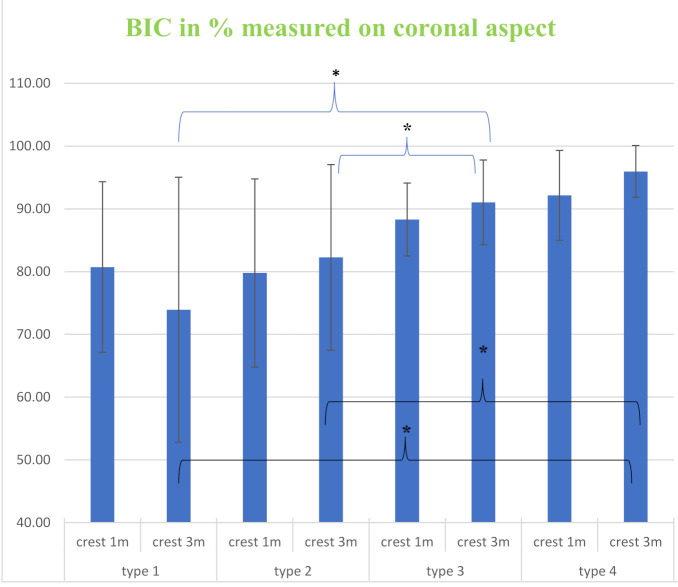
Coronal aspect bone-to-implant contact (BIC) after one month (1 m) and 3 months (3 m) for the four implant types. Data are presented as the average ± sd. * denotes *p* < 0.05.

Healing occurred uneventfully in all animals. The histological sections revealed that all implants were integrated with bone. Furthermore, adaption of the macro- and microscopic design with new bone close to the implant profile (Figure 4) along with blood vessels was observed, with no signs of inflammation; additionally, porous cortical bone was evident. After 1 month of healing, the total bone-to-implant contact (t-BIC) of the four implant types ranged from 85–91% without significant differences among the implant types or between the drilling protocols (Figure 5 and Table 1). After 3 months of healing, the mean t-BIC of implant type 1 decreased from 90% to 75% (DP1) and from 85% to 73% (DP2), while the mean t-BIC of implant types 2–4 slightly increased to 83–84%, 91–92%, and 91–96%, respectively, to implant type, without significant differences between the two drilling protocols (Table 1). At 1 month, the mean coronal BIC of implant type 4 was higher than that of the other implant types. However, there were no statistically significant differences between any of the implant types. After three months of healing, the mean coronal BIC values of implant types 2, 3, and 4 increased. The mean coronal BIC values of implant types 3 (91%) and 4 (96%), both implant types with 2 coronal flutes, were significantly higher than those of implants types 1 (74%) and 2 (82%), both implant types with 6 coronal flutes (Figure 6 and Table 2). Within a fixed drilling protocol, no differences among implant types could be found, possibly due to the low sample size.

## 4. Discussion

The present study investigated the effect of the drilling protocol and implant neck design on the BIC. Two drilling protocols were evaluated: in DP1, implants were inserted into osteotomies smaller than the implant diameter by 0.95 mm; whereas in DP2, implants were inserted into osteotomies smaller than the implant diameter by 0.55 mm, according to the manufacturer’s instructions. The measured BIC of implants inserted according to DP1 and DP2 was comparable for all of the implant types after both 1 m and 3 m of healing for each drilling protocol and ranged from 75–96% after 3 m without significant differences among the implant types or between the drilling protocols. The reported BIC is in agreement with that in a study conducted by Kwon, who reported that the BIC of implants inserted in minipigs ranged from 71–82% after 1 m of healing [32].

Undersized drilling protocols are commonly used for increasing implant primary stability. Such protocols were initially proposed for use in low-density bone, especially in the posterior maxilla [33,34] to enhance the initial implant contact with the bone, optimize bone density, and consequently improve the insertion torque [35]. Conflicting results have been published regarding the effect of these protocols on the implant BIC. In a rabbit tibia model, the insertion of implants with an overdrilling protocol of the crestal aspect of the osteotomy resulted in increased short-term crestal bone-to-implant contact compared to underdrilling [16]. Recently, it was reported that the BIC decreased with overpreparation of the osteotomy site. The initial BIC of implants inserted in undersized osteotomies was the greatest, while the lowest BIC was found in the oversized group [36]. The differences between the two studies may be attributed to the different implant systems, the different sites of implant insertion, and the different drilling protocols, the latter two of which were tibia vs condyle and 0.1 mm vs 0.25 mm overpreparation, respectively. In the current study, the undersized drilling protocol did not have any significant effect on the BIC. Implants inserted according to DP1 and DP2 were comparable for any implant type at both healing times. This probably stems from the soft parietal bone with porous cortical bone into which the implants were inserted.

Four types of implants with different neck microthread and flute designs were evaluated. To the best of our knowledge it is the first study that evaluated the effect of flutes incorporation in implants neck on BIC and it is the first study evaluating two types of microthreads design in-vivo. Regarding the inclusion of flutes in the implant neck, for both microthread types (implants 2 and 3 and implants 1 and 4), the addition of flutes to the coronal aspect resulted in significantly lower BIC values at 3 m. The BIC of implant type 1, with 6 coronal flutes, decreased from 1 m of healing to 3 m, while the BIC of implant type 4, with 2 coronal flutes, slightly increased. A similar trend was observed with implant types 2 and 3. The coronal BIC values of both implant types with 6 coronal flutes did not differ from each other and were significantly lower than those of implant types 3 and 4. The lower BIC values measured for implant types with 6 flutes may stem from excessive stress located at the coronal aspect of the implant. By introducing flutes into the coronal aspect, the implant surface area that can distribute the stress associated with implant insertion decreases dramatically, and the remaining implant surface that is in direct contact with the osteotomy walls is subjected to excessive stress. It has been previously reported that excessive stress is associated with bone resorption [21,22]. To distribute stress at the coronal aspect, microthreads were introduced [23]. Two types of microthreads were compared: threads 0.05 mm in depth (implant types 1 and 4) and threads 0.3 mm in depth (implant types 2 and 3). The coronal BIC of implant type 3, with no coronal flutings, was on average lower than the coronal BIC of implant type 4 at 1 m and at 3 m. This statistically insignificant trend was discerned although implant type 2 has coronal fluting (for implant type 4, coronal flutes compromise approximately 10% of the coronal surface area). As stated above, flutes at the coronal aspect are suspected to impair the coronal BIC, and since implant type 4 presented a higher BIC despite the presence of flutes at the coronal aspect, it can be speculated that the higher number of microthreads of implant type 4 improved the stress distribution and contributed to the higher BIC of implant type 4 compared to that of implant type 3. This notion is supported by Lee et al. [37], who suggested that microthreads increase the implant surface area. Since the microthreads of both implant types are similar in pitch, decreasing the microthread depth enables the production of a greater number of microthreads in a confined area, leading to a greater surface area. Thus, by increasing the surface area, better stress distribution is achieved, resulting in a higher BIC [38]. This is further supported by a recent finite element analysis concluding that the most effective parameters of stress distribution were the depth and pitch of the microthreads [39].

### Limitations

The main limitations of the current study are the vast implant design variations, which included both flutes and microthreads, and the investigation of unloaded implants inserted in calvarial bone, resulting in inconclusive results regarding the superior microthread design. Further studies with a single implant design variation and testing of loaded and unloaded implants should be conducted in variable models including, cad-cam assisted, stem cell, and systemic patients [40,41,42].

## 5. Conclusions

Within the limitations of the current study, the following may be concluded:

The BIC was not affected by the drilling protocol.
Flutes located at the implant coronal aspect significantly impaired the crestal BIC.
Multiple shallow microthreads were associated with a greater crestal BIC.
Further studies should be conducted to confirm the above findings.

## Figures and Tables

**Table 1:** Total bone-to-implant contact (BIC) values of implants types 1–4 inserted with the standard drilling protocol (DP2) and the extra underdrilling protocol (DP1). Values after 1 month and 3 months of healing are presented as the average (%) ± sd.

		Implant Type 1	Implant Type 2	Implant Type 3	Implant Type 4
1 month	DP1	89.47 ± 4.87	80.03 ± 7.26	88.92 ± 5.69	90.58 ± 3.34
	DP2	84.73 ± 7.39	79.64 ± 19.16	88.90 ± 5.06	90.78 ± 6.38
3 months	DP1	74.66 ± 12.93	83.68 ± 8.59	91.02 ± 8.05	91.31 ± 4.38
	DP2	72.78 ± 18.28	82.93 ± 15.27	92.41 ± 4.47	96.17 ± 2.26

**Table 2:** Coronal aspect bone-to-implant contact (BIC) after one month (1 m) and 3 months (3 m) of healing. Values after 1 month and 3 months of healing are presented as the average (%) ± sd.

	Implant Type 1	Implant Type 2	Implant Type 3	Implant Type 4
1 month	80.73 ± 13.60	79.78 ± 14.99	88.30 ± 5.81	92.15 ± 6.21
3 months	73.92 ± 21.11	82.27 ± 14.79	91.04 ± 6.75	95.96 ± 4.13

## Data Availability

The data presented in this study are available on request from the corresponding author.

## Abbreviations

BIC	bone-to-implant contact
t-BIC	total-BIC
c-BIC	coronal-BIC
CFs	coronal flutes
SMs	shallow microthreads
DMs	deep microthreads
DP1	Drilling protocol 1
DP2	Drilling protocol 2
MT	Microthread
T	Thread
FZ	flute zone

## References

[1] Giudice A., Bennardo F., Antonelli A., Barone S., Wagner F., Fortunato L., Traxler H. (2020). Influence of clinician’s skill on primary implant stability with conventional and piezoelectric preparation techniques: An ex-vivo study. J. Biol. Regul. Homeost. Agents.

[2] Szmukler-Moncler S., Piattelli A., Favero G.A., Dubruille J.H. (2000). Considerations preliminary to the application of early and immediate loading protocols in dental implantology. Clin. Oral Implants Res..

[3] Fernandes M., Fonseca E. (2017). Thermal analysis in drilling of ex vivo bovine bones. J. Mech. Med. Biol..

[4] Trisi P., Perfetti G., Baldoni E., Berardi D., Colagiovanni M., Scogna G. (2009). Implant micromotion is related to peak insertion torque and bone density. Clin. Oral Implants Res..

[5] Trisi P., De Benedittis S., Perfetti G., Berardi D. (2011). Primary stability, insertion torque and bone density of cylindric implant ad modum Branemark: Is there a relationship? An in vitro study. Clin. Oral Implants Res..

[6] Marquezan M., Osório A., Sant’Anna E., Souza M.M., Maia L. (2012). Does bone mineral density influence the primary stability of dental implants? A systematic review. Clin. Oral Implants Res..

[7] Tabassum A., Meijer G.J., Wolke J.G., Jansen J.A. (2010). Influence of surgical technique and surface roughness on the primary stability of an implant in artificial bone with different cortical thickness: A laboratory study. Clin. Oral Implants Res..

[8] Tabassum A., Meijer G.J., Wolke J.G., Jansen J.A. (2009). Influence of the surgical technique and surface roughness on the primary stability of an implant in artificial bone with a density equivalent to maxillary bone: A laboratory study. Clin. Oral Implants Res..

[9] Berglundh T., Abrahamsson I., Lang N.P., Lindhe J. (2003). De novo alveolar bone formation adjacent to endosseous implants. Clin. Oral Implants Res..

[10] Turkyilmaz I., Aksoy U., McGlumphy E.A. (2008). Two alternative surgical techniques for enhancing primary implant stability in the posterior maxilla: A clinical study including bone density, insertion torque, and resonance frequency analysis data. Clin. Implant Dent. Relat. Res..

[11] Bashutski J.D., D’Silva N.J., Wang H.L. (2009). Implant compression necrosis: Current understanding and case report. J. Periodontol..

[12] O’Sullivan D., Sennerby L., Meredith N. (2000). Measurements comparing the initial stability of five designs of dental implants: A human cadaver study. Clin. Implant Dent. Relat. Res..

[13] Duyck J., Roesems R., Cardoso M.V., Ogawa T., De Villa Camargos G., Vandamme K. (2015). Effect of insertion torque on titanium implant osseointegration: An animal experimental study. Clin. Oral Implants Res..

[14] Coelho P.G., Marin C., Teixeira H.S., Campos F.E., Gomes J.B., Guastaldi F., Anchieta R.B., Silveira L., Bonfante E.A. (2013). Biomechanical evaluation of undersized drilling on implant biomechanical stability at early implantation times. J. Oral Maxillofac. Surg..

[15] Trisi P., Todisco M., Consolo U., Travaglini D. (2011). High versus low implant insertion torque: A histologic, histomorphometric, and biomechanical study in the sheep mandible. Int. J. Oral Maxillofac. Implants.

[16] Cohen O., Ormianer Z., Tal H., Rothamel D., Weinreb M., Moses O. (2016). Differences in crestal bone-to-implant contact following an under-drilling compared to an over-drilling protocol. A study in the rabbit tibia. Clin. Oral Investig..

[17] Steigenga J.T., Al-Shammari K.F., Nociti F.H., Misch C.E., Wang H.L. (2003). Dental implant design and its relationship to long-term implant success. Implant Dent..

[18] Baumgart F.W., Cordey J., Morikawa K., Perren S.M., Rahn B.A., Schavan R., Snyder S. (1993). AO/ASIF self-tapping screws (STS). Injury.

[19] Bickley M.B., Hanel D.P. (1998). Self-tapping versus standard tapped titanium screw fixation in the upper extremity. J. Hand Surg. Am..

[20] Wu S.W., Lee C.C., Fu P.Y., Lin S.C. (2012). The effects of flute shape and thread profile on the insertion torque and primary stability of dental implants. Med. Eng. Phys..

[21] Hansson S., Werke M. (2003). The implant thread as a retention element in cortical bone: The effect of thread size and thread profile: A finite element study. J. Biomech..

[22] Frost H.M. (1990). Skeletal structural adaptations to mechanical usage (SATMU): 2. Redefining Wolff’s law: The remodeling problem. Anat. Rec..

[23] Chowdhary R., Halldin A., Jimbo R., Wennerberg A. (2015). Influence of micro threads alteration on osseointegration and primary stability of implants: An FEA and in vivo analysis in rabbits. Clin. Implant Dent. Relat. Res..

[24] Schrotenboer J., Tsao Y.P., Kinariwala V., Wang H.L. (2008). Effect of microthreads and platform switching on crestal bone stress levels: A finite element analysis. J. Periodontol..

[25] Metzler P., von Wilmowsky C., Stadlinger B., Zemann W., Schlegel K.A., Rosiwal S., Rupprecht S. (2013). Nano-crystalline diamond-coated titanium dental implants—A histomorphometric study in adult domestic pigs. J. Craniomaxillofac. Surg..

[26] Freilich M., Wen B., Shafer D., Schleier P., Dard M., Pendrys D., Ortiz D., Kuhn L. (2012). Implant-guided vertical bone growth in the mini-pig. Clin. Oral Implants Res..

[27] Romero-Ruiz M.M., Gil-Mur F.J., Ríos-Santos J.V., Lázaro-Calvo P., Ríos-Carrasco B., Herrero-Climent M. (2019). Influence of a novel surface of bioactive implants on osseointegration: A comparative and histomorfometric correlation and implant stability study in minipigs. Int. J. Mol. Sci..

[28] Kilkenny C., Browne W.J., Cuthill I.C., Emerson M., Altman D.G. (2010). Improving bioscience research reporting: The ARRIVE guidelines for reporting animal research. PLoS Biol..

[29] Bosshardt D.D., Bergomi M., Vaglio G., Wiskott A. (2008). Regional structural characteristics of bovine periodontal ligament samples and their suitability for biomechanical tests. J. Anat..

[30] Danz J.C., Habegger M., Bosshardt D.D., Katsaros C., Stavropoulos A. (2014). Virtual tissue alignment and cutting plane definition--a new method to obtain optimal longitudinal histological sections. J. Anat..

[31] Janner S.F.M., Bosshardt D.D., Cochran D.L., Chappuis V., Huynh-Ba G., Jones A.A., Buser D. (2017). The influence of collagen membrane and autogenous bone chips on bone augmentation in the anterior maxilla: A preclinical study. Clin. Oral Implants Res..

[32] Kwon Y.S., Namgoong H., Kim J.H., Cho I.H., Kim M.D., Eom T.G., Koo K.T. (2013). Effect of microthreads on removal torque and bone-to-implant contact: An experimental study in miniature pigs. J. Periodontal. Implant Sci..

[33] Venturelli A. (1996). A modified surgical protocol for placing implants in the maxillary tuberosity: Clinical results at 36 months after loading with fixed partial dentures. Int. J. Oral Maxillofac. Implants.

[34] Martinez H., Davarpanah M., Missika P., Celletti R., Lazzara R. (2001). Optimal implant stabilization in low density bone. Clin. Oral Implants Res..

[35] Bilhan H., Geckili O., Mumcu E., Bozdag E., Sünbüloğlu E., Kutay O. (2010). Influence of surgical technique, implant shape and diameter on the primary stability in cancellous bone. J. Oral Rehabil..

[36] Huang H.M., Chee T.J., Lew W.Z., Feng S.W. (2020). Modified surgical drilling protocols influence osseointegration performance and predict value of implant stability parameters during implant healing process. Clin. Oral Investig..

[37] Lee D.W., Choi Y.S., Park K.H., Kim C.S., Moon I.S. (2007). Effect of microthread on the maintenance of marginal bone level: A 3-year prospective study. Clin. Oral Implants Res..

[38] Abrahamsson I., Berglundh T. (2006). Tissue characteristics at microthreaded implants: An experimental study in dogs. Clin. Implant Dent. Relat. Res..

[39] Geramizadeh M., Katoozian H., Amid R., Kadkhodazadeh M. (2018). Three-dimensional optimization and sensitivity analysis of dental implant thread parameters using finite element analysis. J. Korean Assoc. Oral Maxillofac. Surg..

[40] Cattoni F., Teté G., Calloni A.M., Manazza F., Gastaldi G., Capparè P. (2019). Milled versus moulded mock-ups based on the superimposition of 3D meshes from digital oral impressions: A comparative in vitro study in the aesthetic area. BMC Oral Health.

[41] Capparè P., Tetè G., Sberna M.T., Panina-Bordignon P. (2020). The Emerging Role of Stem Cells in Regenerative Dentistry. Curr. Gene Ther..

[42] Tecco S., Parisi M.R., Gastaldi G., Polizzi E., D’Amicantonio T., Zilocchi I., Gardini I., Gherlone E.F., Lazzarin A., Capparè P. (2019). Point-of-care testing for hepatitis C virus infection at an Italian dental clinic: Portrait of the pilot study population. New Microbiol..

